# A case of pulmonary tuberculosis presenting as diffuse alveolar haemorrhage: is there a role for anticardiolipin antibodies?

**DOI:** 10.1186/1471-2334-10-33

**Published:** 2010-02-20

**Authors:** Almerico Marruchella, Angela Corpolongo, Chiara Tommasi, Francesco N Lauria, Pasquale Narciso

**Affiliations:** 1 Respiratory Endoscopy Unit, National Institute for Infectious Diseases "L. Spallanzani", Via Portuense 292, 00149 Rome, Italy; 2 Clinical Department, National Institute for Infectious Diseases "L. Spallanzani", via Portuense 292, 00149 Rome, Italy

## Abstract

**Background:**

Diffuse alveolar haemorrhage (DAH) has been rarely reported in association with pulmonary infections.

**Case Presentation:**

We report the case of a 43 year old immunocompetent man presenting with dyspnoea, fever and haemoptysis. Chest imaging showed bilateral ground glass opacities. Microbiological and molecular tests were positive for *Mycobacterium tuberculosis *and treatment with isoniazid, rifampicin, ethambutol and pyrazinamide was successful. In this case the diagnosis of DAH relies on clinical, radiological and endoscopic findings. Routine blood tests documented the presence of anticardiolipin antibodies. In the reported case the diagnostic criteria of antiphospholipid syndrome were not fulfilled.

**Conclusions:**

The transient presence of anticardiolipin antibodies in association with an unusual clinical presentation of pulmonary tuberculosis is intriguing although a causal relationship cannot be established.

## Background

Diffuse alveolar haemorrhage (DAH) is a clinico-pathologic syndrome defined by bleeding from alveolar vessels. It may be encountered in many different settings, especially in autoimmune diseases and systemic small-vessel vasculitides, and may be linked to various histological patterns. Pulmonary infections have been rarely associated to DAH. The diagnosis relies upon clinical manifestations, chest imaging, laboratory findings and bronchoalveolar lavage (BAL) [1].

To our knowledge, pulmonary tuberculosis (TB) has been reported only once as the cause of DAH, following autologous stem cell transplantation for diffuse large B cell lymphoma [2].

We report a case of culture proven pulmonary TB presenting as DAH in an immunocompetent man without other risk factors. Patient's written consent was obtained for the case report to be published.

## Case presentation

A 43 year old non-smoking man was admitted in June 2008 because of rapidly progressive exertional dyspnoea, fever and haemoptysis for one week. He reported three cases of pulmonary TB in his family (his father, a sister and an uncle). Past medical history was remarkable only for arterial hypertension, well controlled with amlodipin. He denied exposure to gases, fumes or toxic chemicals. He had never taken any illicit drug in the past.

**Figure 1:**
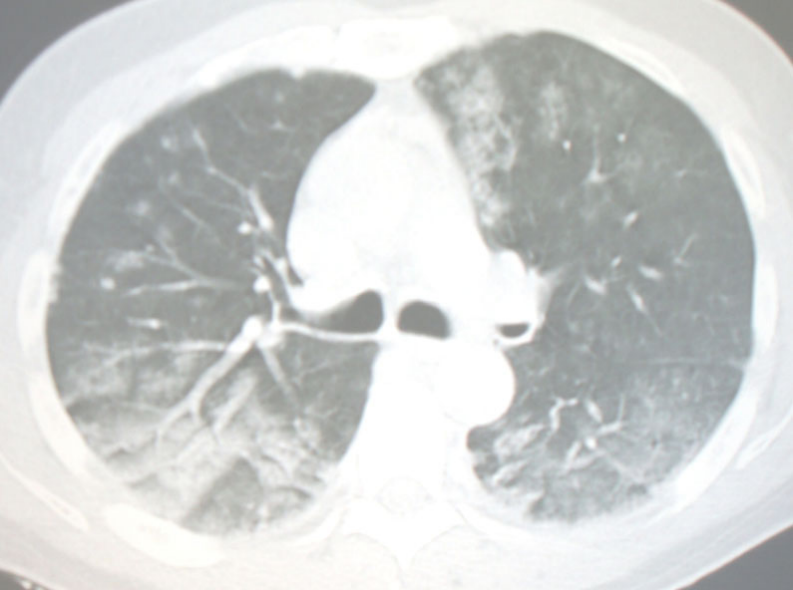
**Chest CT scan at the level of main carina, showing bilateral areas of increased attenuation with a prevalent pattern of ground glass opacities**. Scattered micronodules can be observed.

At admission the patients was dyspnoeic with 30 breaths/min. Chest examination revealed fine bilateral rales. Physical examination of the heart revealed a regular tachycardia (110 beats/min) with normal heart sounds and no murmurs. Arterial blood pressure was 135/90 mmHg. Abdominal findings were normal. There were no signs of lower extremity deep venous thrombosis. Wells score was -2, rendering the diagnosis of venous thromboembolism very unlikely. Chest CT scan showed bilateral areas of increased attenuation with a prevalent pattern of ground glass opacities; focal areas of consolidation and scattered micronodules could be observed (Figure 1). We did not find upper lobe nodules or cavities.

Routine blood tests showed normocromic normocytic anaemia (Hb 11.1 g/dl) with a normal platelet count (118·10^3^/mm^3^; reference range 80-400·10^3^/mm^3^). Coagulation function was normal (aPTT 34 sec, PT 84%, INR 1.13, fibrinogen 559 mg/dl). Urinalysis was unremarkable. ESR 41 mm/h (reference value <15). Auto antibodies (ANA, ENA, AGBM, anti-dsDNA and ANCA) and the lupus anticoagulant test were negative. Anticardiolipin (aCL) IgG antibodies were found (76 UGPL; reference value <13) while anti-β2-glycoprotein-I (β2-GPI) antibodies were absent. Tuberculin skin test (5 U PPD) was negative such as a T-cell INF-γ release assay for TB infection (QuantiFERON-TB gold: 0.10 UI/ml; cut-off value 0.35 UI/ml). Screening test for HIV-Ab was non reactive. Arterial blood gases breathing room air revealed hypoxemia (66 mmHg) and hypocapnia (31 mmHg).

Flexible bronchoscopy (FBS) showed fresh blood in all segmental bronchi. BAL in the left upper lobe obtained a progressively bloodier return in the five 20 ml aliquots. Microscopy revealed red blood cells and alveolar macrophages (90% of total cells, lymphocytes and neutrophils accounting for 4% and 6% respectively). About 40% of alveolar macrophages had cytoplasmic haemosiderin inclusions (iron staining). No tissue biopsies were taken.

Bacterioscopic examination of BAL and sputum were negative for acid fast bacilli such as culture for common bacteria, while molecular test for *Mycobacterium tuberculosis *(16S rRNA) gave a positive result in a few hours, so that a standard therapy with isoniazid, rifampicin, ethambutol and pyrazinamide was started on the same day. Corticosteroids were not added to the therapy. Fever, cough and haemoptysis resolved in the first week while chest X-ray cleared in about 4 weeks. Cultures grew *M. tuberculosis *in 21 days and drug susceptibility test did not show any resistance to first line drugs.

After 8 weeks, anaemia resolved (Hb 14.5 g/dl) and aCL antibodies became negative. Ethambutol and pyrazinamide were discontinued, and isoniazid and rifampicin were continued for 16 weeks.

## Conclusions

DAH has been reported in many clinical settings but is often associated with autoimmune diseases or systemic small-vessel vasculitides. Rarely drugs and chemicals have been involved in the pathogenesis of DAH [1]. Among pulmonary infections, there are reports relating DAH to viruses, *mycoplasma*, and *legionella *[3-5]. Pulmonary TB has been reported in a patient presenting with DAH following autologous bone marrow transplantation. TB was diagnosed *post-mortem *on BAL culture [2].

In this case the diagnosis of DAH relies on clinical (haemoptysis, anaemia), radiological, endoscopic and laboratory findings. On the other hand, the diagnosis of pulmonary TB was established by molecular tests and culture of BAL fluid and the causal link between TB and DAH was strongly supported by the prompt clinical response to anti-mycobacterial therapy. The negative results of PPD and QuantiFERON-TB confirm the low sensitivity of these tests in active TB making them of limited clinical usefulness [6].

Antiphospholipid (aPL) antibodies have been found in association with many infectious diseases: viral (HCV, HBV, HIV, CMV, EBV, parvovirus B19, HTLV-1), bacterial and mycobacterial (leprosy, syphilis, rickettsiosis, leptospirosis), and parasitic (malaria, kala-azar) infections. In particular Elkayam et al found aCL IgG and IgM in a proportion of TB patients significantly higher than in normal controls [7]. In these circumstances aCL antibodies are usually not associated with anti-β2-GPI antibodies and clinical findings of antiphospholipid syndrome (thrombotic and haematological). On the other hand DAH has been described in patients with antiphospholipid syndrome (APS) [8]. In this case the diagnostic criteria of APS were not fulfilled. However, the transient presence of aCL antibodies in association with an unusual clinical presentation of TB is intriguing, although a causal relationship cannot be established.

To our knowledge this is the first reported case of pulmonary TB associated with a clinical picture of DAH in an immunocompetent patient without other risk factors. We suggest that an infectious cause should always be searched for in the diagnostic work-up of DAH because of obvious therapeutic implications. Molecular tests for *M. tuberculosis *may be of value, allowing for a timely diagnosis and prompt therapy. The prevalence and possible pathogenic role of aPL antibodies in patient with TB should be explored in a prospective manner.

## Abbreviations

ANA: antinuclear antibodies; ENA: extractable nuclear antigens; AGBM: anti-glomerular basement membrane; ANCA: anti-neutrophil cytoplasmic antibodies; HCV: hepatitis C virus; HBV: hepatitis B virus; HIV: human immunodeficiency virus; CMV: cytomegalovirus; EBV: Epstein-Barr virus; HTLV-1: human T-lymphotropic virus-1.

## Competing interests

The authors declare that they have no competing interests.

## Authors' contributions

AM (pulmonologist) performed FBS and BAL and drafted the manuscript. AC, CT and PN (infectious diseases specialists) were the reference physicians during the in-hospital management of this case. FNL (infectious diseases specialist) was the reference physician during the out-patient phase of management.

All authors contributed to the article drafting, read and approved the final manuscript.

## Pre-publication history

The pre-publication history for this paper can be accessed here:

http://www.biomedcentral.com/1471-2334/10/33/prepub
